# Wearable Electronics of Silver-Nanowire/Poly(dimethylsiloxane) Nanocomposite for Smart Clothing

**DOI:** 10.1038/srep13971

**Published:** 2015-09-24

**Authors:** Gui-Wen Huang, Hong-Mei Xiao, Shao-Yun Fu

**Affiliations:** 1 Technical Institute of Physics and Chemistry, Chinese Academy of Sciences, No.2 Beiyitiao, Zhongguancun, Beijing, 100190, P. R. China; 2 University of Chinese Academy of Sciences, Beijing 100039, China

## Abstract

Wearable electronics used in smart clothing for healthcare monitoring or personalized identification is a new and fast-growing research topic. The challenge is that the electronics has to be simultaneously highly stretchable, mechanically robust and water-washable, which is unreachable for traditional electronics or previously reported stretchable electronics. Herein we report the wearable electronics of sliver nanowire (Ag-NW)/poly(dimethylsiloxane) (PDMS) nanocomposite which can meet the above multiple requirements. The electronics of Ag-NW/PDMS nanocomposite films is successfully fabricated by an original pre-straining and post-embedding (PSPE) process. The composite film shows a very high conductivity of 1.52 × 10^4^ S cm^−1^ and an excellent electrical stability with a small resistance fluctuation under a large stretching strain. Meanwhile, it shows a robust adhesion between the Ag-NWs and the PDMS substrate and can be directly machine-washed. These advantages make it a competitive candidate as wearable electronics for smart clothing applications.

Wearable electronics for smart garments or accessories, a new and fast-growing multidirectional research area, has necessitated the demand for flexible and stretchable conductors^1^,^2^,^3^,^4^,^5^. As a result of human body movements, these electronics embedded in clothes will be stretched, folded, twisted or crumpled^6^,^7^,^8^,^9^. Furthermore, they also need to be washed together with clothes^10^. It requires that the electronics can endure these operations without degradations in performances. Namely, the wearable electronics should meet the requirements of being simultaneously stretchable, robust and water-washable^6^,^11^,^12^,^13^. However, it is hard for electronics to simultaneously meet these requirements. Works on electronics which own one of the advantages have been carried out^14^,^15^,^16^, but rarely combinative results have been reported yet. Therefore, in this work we will focus on developing a wearable electronics that can meet all the above multiple requirements.

In order to obtain stretchable electronics with high electrical stability, a great deal of research work has been carried out based on conductive thin films such as conductive polymer composite films^17^, metal films with a wavy geometry^18^,^19^ or hybrid films based on carbon nanotubes and graphene^20^,^21^,^22^,^23^. Nevertheless, dramatic electrical resistance rise has been observed under a large stretching strain. For example, an increase of >100% over the initial value was noticed under the 80% stretching strain^17^,^18^,^20^. In other words, the electrical stability is not satisfactory for these previously reported conductive film circuits. Alternatively, one-dimensional metallic nanowires have been used as conductive materials to prepare electric circuits based on stretchable substrates since their high aspect ratio can afford high electrical conductivity and mechanical compliance^14^,^24^. Among metal nanowires, silver nanowires (Ag-NWs) are considered as the most promising choice for stretchable circuits since silver posses the highest electrical conductivity among metals and Ag-NWs are known to have superior yield strength and Young’s modulus over bulk silver^25^,^26^. Due to their outstanding performance, a number of interesting applications based on Ag-NWs have been demonstrated in recent years, including flexible organic light emitting diodes with Ag-NW transparent conductors^27^,^28^, flexible transparent conductors and touch panels^29^,^30^,^31^,^32^,^33^, flexible fuel cells^34^,^35^, pH-triggered electrical switches^36^, paper-based flexible circuits^37^, conducting adhesives^38^, and so on. Furthermore, several Ag-NW electric circuits based on stretchable substrates have been reported and reasonable electrical stability has been demonstrated^14^,^15^. For instance, Lee *et al.*^14^ fabricated a highly stretchable Ag-NW conductor based on a pre-strained stretchable elastomer (Ecoflex) substrate and achieved a good circuit electrical stability under large stretching strains. In the circuit, Ag-NW network was deposited on the top of the Ecoflex substrate. Due to the very soft nature of Ecoflex, Ag-NW network could be stuck to the substrate at moderate areal density. However, when the Ag-NW areal density was high, it behaved like a thin film and might generate cracks and became detached from the substrate, which would limit the content of Ag-NWs used in the circuit^14^. On the other hand, Zhu *et al.*^16^ reported a stretchable conductor with Ag-NWs embedded in the surface layer of poly(dimethylsiloxane) (PDMS) using an embedding process. A strong adhesion of Ag-NWs to the PDMS substrate was obtained due to the embedding of Ag-NWs within the PDMS. Nonetheless, the electrical stability of the conductors under stretching was relatively low since the embedded Ag-NW networks could be readily damaged upon a high stretching deformation.

As both the high electrical stability and good mechanical robustness are highly desired for wearable circuits^6^,^10^,^11^,^14^,^15^,^16^, it is very important to simultaneously achieve these abilities. In this study, highly stretchable, electrically stable and mechanically robust electronics are developed from the Ag-NW/PDMS nanocomposites using the pre-straining and post-embedding (PSPE) process. PDMS is chosen as the substrate because on the one hand it owns a high stretchability and on the other hand it is waterproof which can make the embedded electronics water-washable. The advantages of the pre-staining method and the embedding approach are integrated into the PSPE process. Indeed, the as-prepared PSPE circuits show both high electrical stability and excellent mechanical robustness. A small resistance fluctuation and an excellent adhesion of Ag-NWs to the PDMS substrate are observed for the PSPE electronics. Moreover, it can be directly machine-washed together with clothes without degradations in performance. Finally, stretchable and washable radio frequency identification (RFID) tags made by the PSPE process are demonstrated for their successful application in smart garments or accessories.

## Results and Discussion

### Pre-straining and post-embedding fabrication process

**Figure 1:**
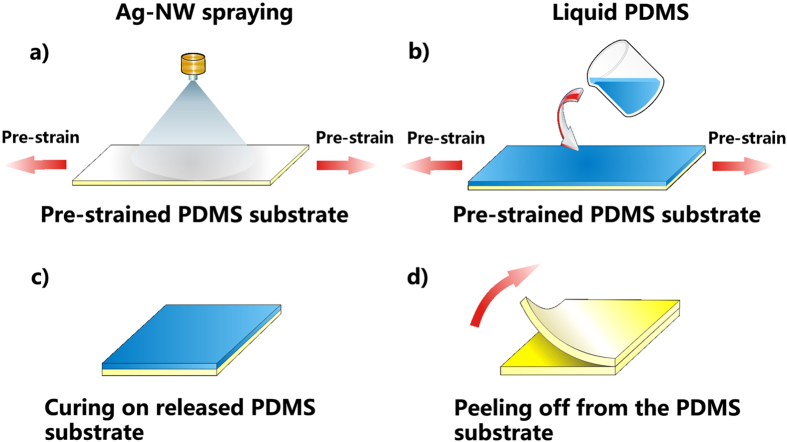
Schematic diagram of the pre-straining and post-embedding process. (**a**) Ag-NW solution is sprayed onto the pre-strained PDMS substrate, (**b**) liquid PDMS is cast on the Ag-NW network formed on the pre-strained PMDS substrate, (**c**) the pre-strained PDMS substrate is released and the liquid PDMS is cured and (**d**) the PSPE circuit is peeled off from the substrate. The author Gui-Wen Huang drew this schematic diagram.

A schematic representation of the PSPE process is shown in Fig. 1. In the PSPE process, a cured PDMS film is used as the substrate under a pre-strain. Initially, the Ag-NW-ethanol suspension is sprayed onto the 80% pre-strained PDMS substrate to form a uniform silver conductive network (Fig. 1a). The 80% pre-strain is the maximum stretching deformation for PDMS^16^. Then, liquid PDMS is cast on the top of the Ag-NW network on the pre-strained PMDS substrate, followed by letting the liquid PDMS completely infiltrate into the Ag-NW network (Fig. 1b). Afterwards, the mechanical pre-strain is released and the Ag-NW network formed in the PMDS is buckled to a wavy structure on the released PDMS substrate. After cured at 150 °C for 30 mins (Fig. 1c), the PSPE circuit is obtained by peeling off it from the released PDMS substrate (Fig. 1d). The Ag-NW network top-surface is exposed *via* carefully controlling the process and the amount of the liquid PMDS. The content of the Ag-NWs in the circuit is described by an areal density (AD, namely the Ag-NWs weight in per unit area of the circuit).

### Conductivity and stretchability of the composite circuits

**Figure 2:**
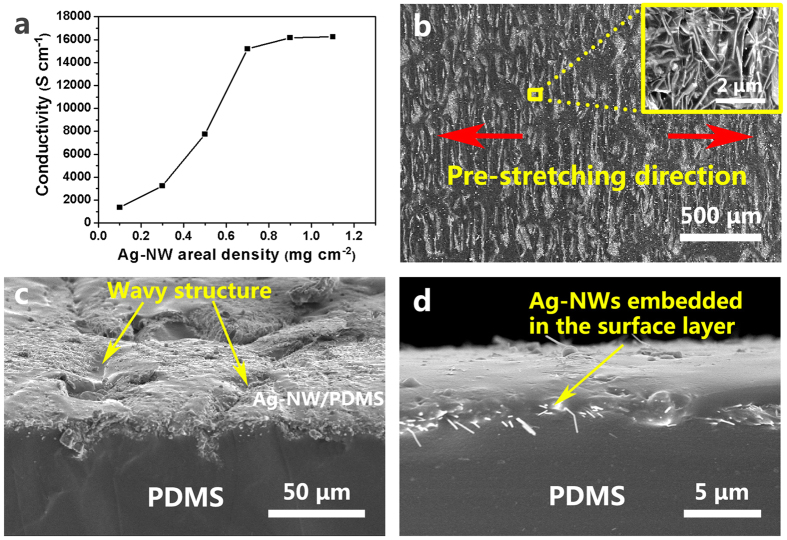
Electrical and morphological characterizations of the composite circuits. (**a**) Conductivity of the PSPE circuit as a function of the Ag-NW areal density, (**b**) top-view SEM image of the surface of the PSPE circuit, the inset shows the magnified image in which the Ag-NWs can be clearly seen, (**c**) side-view SEM image of the surface of the PSPE circuit and (**d**) SEM image of the cross-section of the PSPE circuit.

Figure 2a shows the conductivity of the circuit as a function of the Ag-NW areal density. It can be seen that the percolation threshold appears at the areal density of 0.7 mg cm^−2^ and the corresponding electrical conductivity value is very high (1.52 × 10^4^ S cm^−1^), which is much higher than that of the conductivity (8.13 × 10^3^ S cm^−1^) of the reported stretchable Ag-NW/PDMS circuit^16^. Furthermore, this conductivity can meet the requirements of various electronic applications since it is close to that of universal Sn/Pb eutectic solders (~3 × 10^4^ S cm^−1^)^14^,^39^,^40^,^41^. Therefore, 0.7 mg cm^−1^ of Ag-NWs is selected as the suitable areal density for fabricating the strechable circuits. Figure 2b shows the scanning electronic microscopy (SEM) image of the top surface of the Ag-NW/PDMS nanocomposite circuit with an Ag-NW AD of 0.7 mg cm^−1^, where a wavy pattern can be seen in the stretching direction. The wavy structure is the key factor leading to the high electrical stability of the circuit upon stretching. Figure 2c shows the magnified SEM image of the surface of the circuit and the wavy structure can be clearly seen. Figure 2d exhibits that Ag-NWs are buried in the PDMS surface layer. On the top surface is the Ag-NW/PDMS nanocomposite forming a conductive layer.

Except the PSPE circuit, the Ag-NW/PDMS stretchable circuits are also prepared according to the previously reported pre-straining approach^14^ and the embedding approach^16^. These two techniques were chosen for comparison because they showed reasonable advantages in fabricating Ag-NW stretchable circuits^14^,^16^. The fabrication of the embedded circuits and the pre-strained circuits is described here in brief. For the embedded circuits, the Ag-NWs are initially cast onto a plane glass substrate. After dried, liquid PDMS is cast on the top of the Ag-NW film, followed by curing at 65 °C for 12 h. Finally, the embedded circuit is obtained by peeling off it from the glass substrate^16^. For the pre-strained circuits, Ag-NWs are firstly deposited on a Teflon filter to form a uniform network, which is then transferred to the 80% pre-strained PDMS substrate by applying uniform suction pressure on the other side of the filter. Afterwards, the circuit undergoes a subsequent thermal annealing process in a 220 °C convection oven for 2 h to remove the polyvinylpyrrolidone (PVP) surfactant and allow nanowelding. Here the nanowelding means that the silver nanowires at their contact points are melted at a high temperature and are then joined together, which is helpful for reducing the contact resistance between silver nanowires^14^.

**Figure 3:**
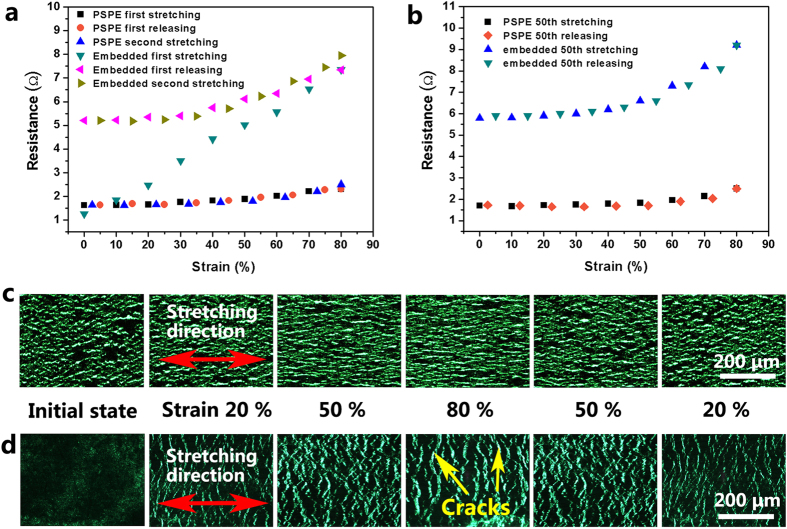
Characterizations of electrical stability during stretching. (**a**) Resistance of the PSPE circuit and the embedded circuit as a function of tensile strain, (**b**) resistance of the PSPE circuit and the embeded circuit as a function of tensile strain at the 50th stretching, (**c**) the transmission mode optical image for the PSPE circuit and (**d**) the transmission mode optical image of the embedded circuit at initial state and under various stretching strains upon stretching and releasing.

Figure 3a shows the resistance of PSPE circuit and the embedded circuit as a function of tensile strain. The silver nanowire AD of the two circuits is 0.7 mg cm^−2^ and they are cut into the same test samples with a size of 1 × 5 cm^2^. It can be seen that for the embedded circuit, during its first stretching, the resistance increases almost linearly from 1.25 Ω to 7.35 Ω when the tensile strain is increased to 80%. Upon releasing of the strain, the resistance is partially recovered and decreased to 5.2 Ω when the substrate is fully released. Namely, a great increase of 488% in resistance occurs during its first stretching and a permanent increase over 300% in resistance is left after the first stretching-releasing cycle. After the first releasing, the embedded circuit is stretched again to 80% strain, an obvious increase in resistance occurs. By contrast, for the PSPE circuit, the increase in the resistance is extremely low only from 1.62 Ω to 2.40 Ω at the tensile strain of 80% during its first stretching. When the strain is released, the resistance is fully recovered with almost no permanent increase left. During the second stretching, the resistance changing tendency is similar to that of the first stretching. Fifty stretching cycles upon the two circuits are performed. Figure 3b shows the resistance of the two circuits at the 50th stretching and releasing cycle. As can be seen, the PSPE circuit maintains a very stable resistance value after 50 stretching cycles with an increase of 0.8 Ω at 80% of tensile strain. On the contrary, a larger resistance increase can be observed for the embedded circuit after 50 stretching cycles. During the 50th cycle, the increase in the resistance has a large value of 3.4 Ω when the substrate is stretched to 80% tensile strain. This indicates that the as-prepared circuit using the present PSPE process has a much better electrical stability compared to the embedded circuit prepared using the embedding process^20^.

To probe the underlying mechanism of the electrical behavior under stretching, the morphology of the Ag-NW network in the circuits is monitored *in situ* during stretching *via* an optical microscopy. The transmission mode optical microscopy images, in which the light source is green, of the PSPE circuit and the embedded circuit corresponding to a series of applied strains during stretching and releasing are respectively displayed in Fig. 3c,d. As the PDMS substrate is optically transparent, the morphology of the Ag-NW networks under strains can be clearly seen. For the PSPE circuit, the stripes in the Ag-NW network are caused by the tensile strain generated during releasing of the pre-strain, which is perpendicular to the pre-strain direction. When the PSPE circuit is stretched, the morphology of the Ag-NW network in stretching direction does not show obvious change. This can be attributed to its wavy structure formed by the pre-straining process. The pre-train of 80% is the maximum tensile strain value of the PDMS substrate^16^. When the circuit is stretched between 0% and 80%, the wavy-structured Ag-NW network will be made gradually flattened, thus no noticeable damage in the conductive network can be observed, leading to a very high electrical stability upon stretching of the PSPE circuit. On the contrary, when the embedded circuit is stretched, the Ag-NW network is torn in the stretching direction as the PDMS substrate deforms and obvious cracks in the network can be clearly seen as marked by yellow arrows in Fig. 3d. These cracks result in the dramatic degradation of the electrical property of the circuit especially under large tensile strains. Therefore, the PSPE circuit demonstrates much higher electrical stability upon stretching than the embedded circuit.

### Adhesion ability between Ag-NWs and PDMS substrate

**Figure 4:**
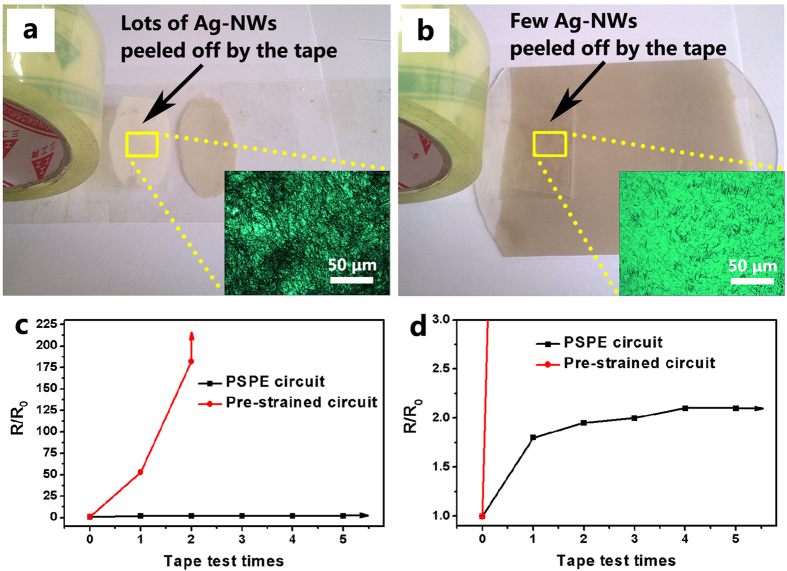
Contrast tape tests. Digital photographs for the tape test for (**a**) the pre-strained circuit and (**b**) the PSPE circuit, the insets are the optical micrographs of the tape surfaces after the first-time testing, (**c**) relative resistance of the two circuits as a function of the tape test times and (**d**) the magnified relative resistance (up to 3) versus tape-test-times curve of the PSPE circuit.

The adhesion ability between circuit and substrate is critically important for stretchable circuits to get practical applications^14^,^15^,^16^. Weak adhesion leads to delamination and failure of the circuits after repeated stretching, which is unacceptable for electrical devices. To examine the adhesion between Ag-NWs and PDMS substrate, a contrast tape test is conducted between the PSPE circuit and the pre-strained circuit as shown in Fig. 4a,b. In this test, the biaxially oriented polypropylene based pressure-sensitive adhesive tapes of primary adhesion ≥13 (measured following the JIS Z0237-2000 standard) are firstly pasted on the top of the circuits under uniform pressure and then peeled off from the surfaces of the circuits. The insets in Fig. 4a,b respectively show the optical micrographs of the tapes after the first tape test on the two circuits. It can be seen that lots of Ag-NWs are adhesively peeled off from the pre-strained circuit after the first tape test while much fewer Ag-NWs are peeled off from the PSPE circuit.

Figure 4c–d shows the relative resistance of the two circuits as a function of the tape test times. The data reveals that the resistance of the pre-strained circuit increases dramatically during testing and becomes infinity after only two tape tests. In comparison, the PSPE circuit exhibits a robust response over 5 times of tape tests with a much lower increase in the electrical resistance, demonstrating the strong adhesion of Ag-NWs to the PDMS substrate for the PSPE circuit. Due to the embedding effect, the majority of the Ag-NWs are buried under the surface of the PDMS for the PSPE circuit, and only few Ag-NWs on the very top of the PDMS surface can be peeled off from the circuit. However, in the pre-strained circuit, all the Ag-NWs are deposited on the surface of the substrate, which can be easily peeled off by external forces.

In order to further demonstrate the advantages of the PSPE process, Table 1 displays the comparison of the electrical stability upon stretching and adhesion of the stretchable circuit in the present work with the reported data of other Ag-NW-based stretchable circuits^14^,^15^,^16^,^42^. It is clear from Table 1 that the electrical stability of the PSPE circuit in the present work under stretching (46% at 80% strain; 8% at 10% strain) is much better than for the embedded PUS/Ag-NW/PDMS circuit (850% at 80% strain), the embedded Ag-NW/PDMS circuit (459% at 80% strain), the pre-strained Ag-NW/Ecoflex circuit (80% at 80% strain) and the biaxially pre-strained Ag-NW/PDMS circuit (42% at 10% strain). Notably, besides the high electrical stability, the PSPE circuit owns strong adhesion of Ag-NWs to the substrate, which makes it a promising competitor in practical applications of stretchable electrics. These two requirements of high electrical stability and excellent adhesion cannot be simultaneously met by any of the previous works^14^,^15^,^16^,^42^ but can be met by the present work.

### Wearable electronics

**Figure 5:**
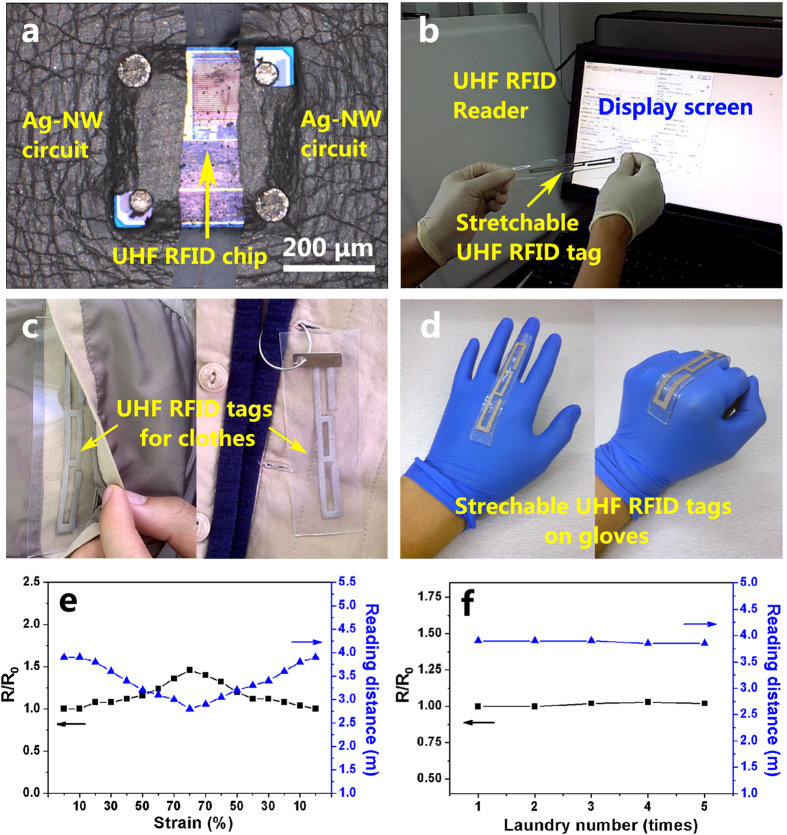
Demonstration of wearable electronics using the composite circuits. (**a**) Optical micrograph for the UHF chip connected to the PSPE circuit, (**b**) reading of the UHF label under stretching strain by a reader, (**c**) the Ag-NW/PDMS embedded UHF RFID tag (left)and hangtag (right) used in clothes, (**d**) the Ag-NW/PDMS stretchable UHF RFID tags on gloves, (**e**) the relative resistivity and the reading distance of the tag as a function of the stretching strain and (**f**) the relative resistivity and the reading distance of the tag as a function of the laundry number, in which each standard laundry process lasts for about 40 min. In (**e**,**f**), R_0_ value equals 6.7 × 10^−7^ Ω·m.

The PSPE process is applied for preparation of the stretchable and washable RFID labels used in smart clothes and accessory systems. The fabrication details are given in Fig. S2 in the electronic supplementary information (ESI). As shown in Fig. 5a, the ultra-high frequency (UHF, 915 MHz) chip is connected to the patterned Ag-NW antenna and is embedded in the PDMS. After cured, the stretchable RFID label is obtained by peeling off it from the substrate. The information in the chip can be easily read during stretching and is exhibited on the display screen as shown in Fig. 5b (see Video 1 in ESI, the details about the chip and equipment are given in ESI). Figure 5c displays the application examples of the RFID labels as embedded and hanging tags in clothes. Figure 5d shows the application of the tags on gloves, where a large strain is created when making a fist. As the PDMS is waterproof, the tags can be directly machine-washed with the clothes as evidenced below and in the ESI (Fig. S4).

Fig. 5e reveals the relative resistivity and the reading distance of the RFID label as a function of the stretching strain. The reading distance is defined as the maximum distance between the RFID label and the reader when the information in the chip of the label can be read by the reader and the equipment setup for the RFID reading experiment is shown in the ESI (Fig. S3). The reading distance of the label is decreased about 25% under the maximum strain of 80% but can still remain about 3 meters of reading distance, which is even better than that of the Ag-NW RFID label under no stretching condition reported in the literature^16^. The relative resistivity and the reading distance of the label as a function of the laundry number are presented in Fig. 5f. The laundry process is conducted using a fully automatic washing machine and each standard laundry process lasts for about 40 min. The details for the laundry experiment are given in the ESI (Fig. S4). Figure 5f shows that the electrical property and the reading distance of the label remain almost constant during 5 times of laundry. The above results indicate that the PSPE process is very promising for fabricating high performance wearable electronics for practical applications.

In summary, washable and stretchable electronics based on Ag-NW/PDMS nanocomposites for smart clothing has been successfully fabricated using the pre-straining and post-embedding (PSPE) process. By combining the advantages of “pre-straining” and “embedding” processes, the PSPE circuit shows an extremely high electrical stability under a large stretching strain while meanwhile owes a robust adhesion of Ag-NWs to the PDMS substrate. The PSPE circuits with a very high conductivity of 1.52 × 10^4^ S cm^−1^ and a small resistance fluctuation of 48% at 80% tensile strain are demonstrated, showing the obvious superiority of the as-developed electronics over the previously reported stretchable circuits. Moreover, it is water-washable and can be directly machine-washed with clothes without obvious degradations in performances. These advantages make it a promising candidate as electronics for smart clothing applications.

## Methods

### Materials

Reagents including AgNO_3_, FeCl_3_·6H_2_O, and ethylene glycol are all in analytical grade and are used as received. These reagents were purchased from Beijing Chemical Works, China. Polyvinylpyrrolidone (PVP) was purchased from Alfa Aesar Company. Dimethyl siloxane and curing agent were purchased from Jonby Electronic Co., Ltd, Nanjing, China.

### Synthesis of silver nanowires

In the synthesis of Ag-NWs, a modified FeCl_3_-mediated process was used. First, 1 × 10^−5^ mol of FeCl_3_·6H_2_O and 1.4 × 10^−2^ mol of PVP were added to 100 ml ethylene (EG) to form additive solution. Second, 1.0 × 10^−2^ mol AgNO_3_ was dissolved in another 100 ml EG to form basic solution. Then, the additive solution was added drop wise to the basic solution at the speed of 5 ml/min under vigorous stirring. Afterwards, the mixture was heated at 160 °C for 3 h in an autoclave for the growth of Ag-NWs. Finally, the Ag-NWs were obtained by rinsing with a large amount of acetone. The synthesized silver nanowires have an average length of 25.7 μm and an average diameter of 187 nm. Details of the size distribution of the Ag-NWs are given in Fig. S1 in ESI.

### Preparation of PDMS

PDMS was prepared by mixing the “base” and the “curing agent” with a ratio of 10:1. After vacuum de-foaming, the liquid mixture was then thermally cured at 150 °C for 30 mins to form cross-linked solid PDMS.

### Structural characterization

Scanning electron microscopy images for composite samples were taken using Hitachi-S 4800 with the 10 kV electron source. Optical microscopy images of Ag-NW networks were taken by Olympus STM6 Optical Microscope. Electrical conductivity of circuits was measured with a Keithley SourceMeter 2400 using the standard four-probe method.

## Additional Information

**How to cite this article**: Huang, G.-W. *et al.* Wearable Electronics of Silver-Nanowire/Poly(dimethylsiloxane) Nanocomposite for Smart Clothing. *Sci. Rep.* **5**, 13971; doi: 10.1038/srep13971 (2015).

## Supplementary Material

Supplementary Information

Supplementary Video 1

## Figures and Tables

**Table 1:** Comparison of relative resistance and adhesion of the PSPE Ag-NW/PDMS stretchable circuit with other circuits fabricated by some existing processes^14^,^15^,^16^,^42^.

**Materials**	**Process**	**∆R/R_0_after stretching**	**Adhesion ability**	**Ref no.**
PUS/Ag-NW/PDMS	Embedding	850% at 80% strain	Strong	42
Ag-NW/PDMS	Embedding	459% at 80% strain	Strong	16
Ag-NW/Ecoflex	Pre-straining	80% at 80% strain	Fair at low content, poor at high content	14
Ag-NW/PDMS	PSPE	46% at 80% strain	Strong	Present work
Ag-NW/PDMS	Biaxial pre-straining	42% at 10% strain	Poor	15
Ag-NW/PDMS	PSPE	8% at 10% strain	Strong	Present work
