# Supplementary material for: Wearable Electronics of Silver-Nanowire/Poly(dimethylsiloxane) Nanocomposite for Smart Clothing
Source: Sci Rep. 2015 Sep 24;5:13971. doi: 10.1038/srep13971 (PMC4585879; doi:10.1038/srep13971)
Supplement: Supplementary Information [file srep13971-s2.pdf]

Supplementary Information

Wearable Electronics of Silver-Nanowire/Poly(dimethylsiloxane) Nanocomposite for Smart Clothing

Gui-Wen Huang[1,2], Hong-Mei Xiao[1] and Shao-Yun Fu[1,*]

[1]Technical Institute of Physics and Chemistry, Chinese Academy of Sciences, No.2 Beiyitiao, Zhongguancun, Beijing, 100190, P. R. China

[2]University of Chinese Academy of Sciences, Beijing 100039, China

*Corresponding to syfu@mail.ipc.ac.cn

Video 1: The stretchable RFID label is read during stretching by a RFID reader.

1. Characterization of synthesized silver nanowires

Figure S1a and **1b** show the SEM images of the as-synthesized silver nanowires (Ag-NWs). The distribution of the diameter and length of the Ag-NWs are given in **Figure S1c** and **1d**, respectively. The average diameter and length of the Ag-NWs are obtained from 100 stochastic measurements in terms of the SEM images of the Ag-NWs using Nano Measure software. The Ag-NWs have an average length of 25.7 μm and an average diameter of 187 nm.

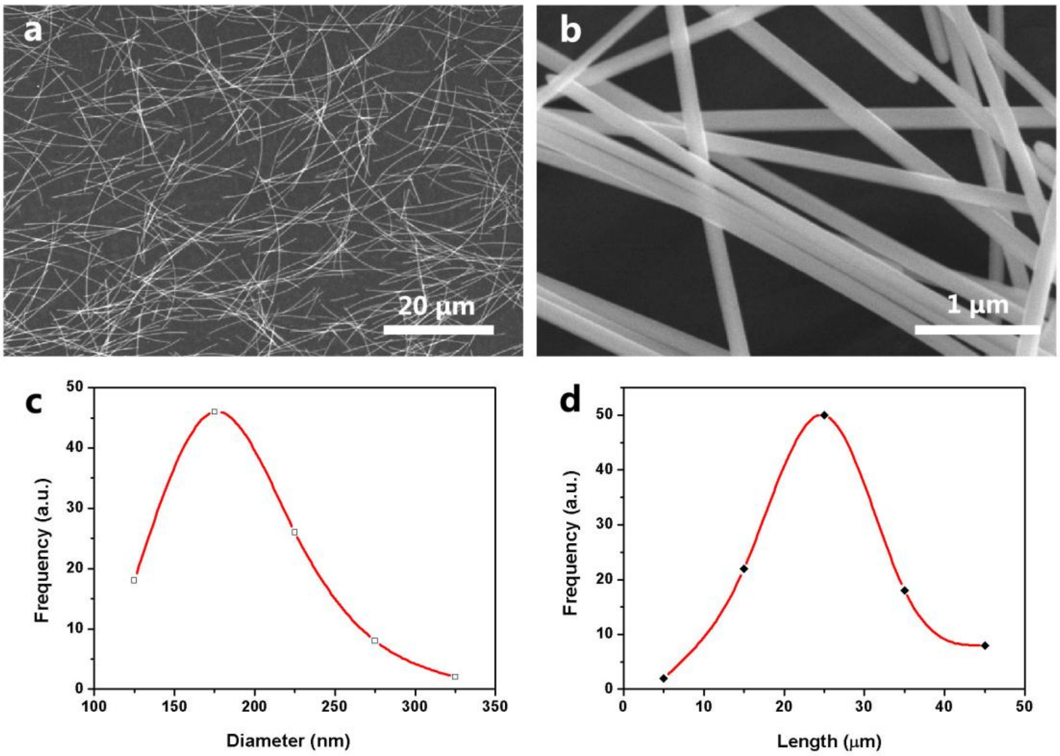

Figure S1. (a) Low resolution SEM image and (b) high resolution SEM image of the Ag-NWs; (c) the diameter distribution and (d) the length distribution of the Ag-NWs.

2. Stretchable and washable RFID label

First, a cured PDMS film is pre-stretched to a strain of 80 % and the pre-strained PDMS film is used as the substrate. Ag-NW-ethanol suspension is then sprayed on the substrate though a film mask to form the patterns of RIFD antenna as shown in **Figure S2a**. Second, RFID chips are placed on the connecting points of the Ag-NW antenna as shown in **Figure S2b**, where the inset shows the magnified optical microscope image of the chip. Then, the liquid PDMS is casted on the antenna to cover the Ag-NWs and chips. Afterwards, the mechanical pre-strain is released and

the antenna and chips are well embedded in the liquid PDMS. The PSE stretchable RFID labels are prepared on the PDMS substrate as displayed in **Figure S2c**. After cured at 150 ℃ for 30 min, the stretchable and washable RFID labels are obtained by peeling off them from the substrate as shown in **Figure S2d**.

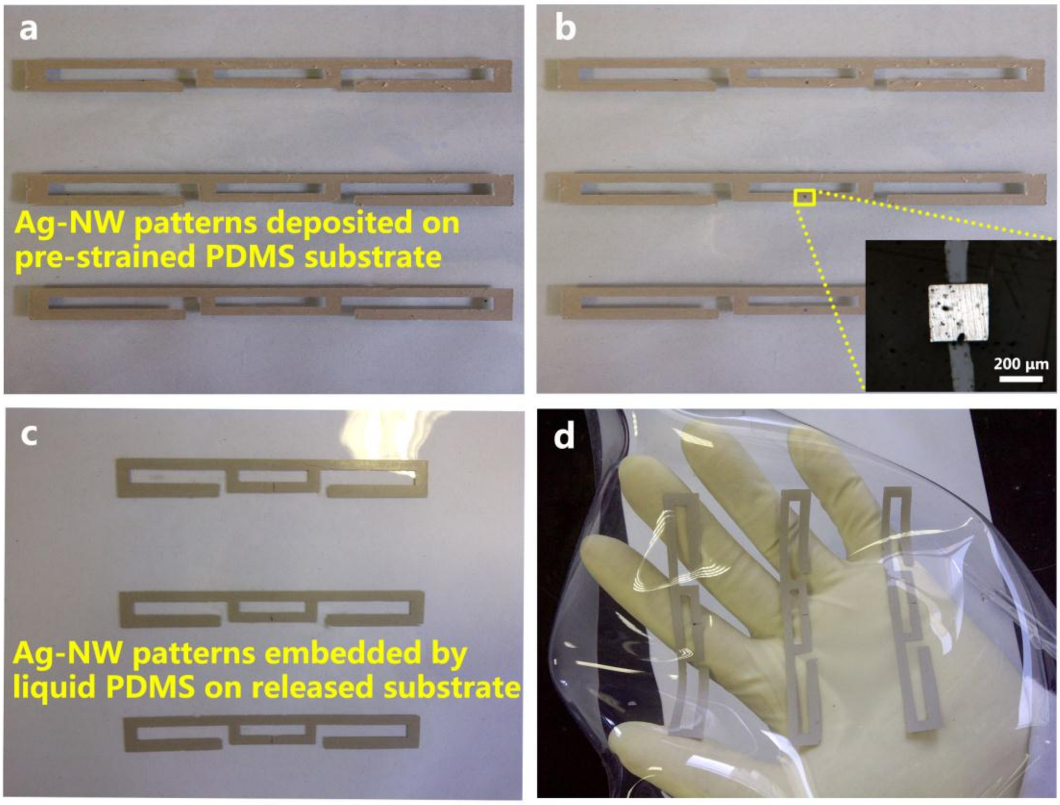

Figure S2. Process of fabricating PSE stretchable RFID label: (a) Ag-NW antenna patterns are sprayed onto the pre-strained PDMS substrate through a film mask, (b) the chips are placed on the antenna, (c) the liquid PDMS is casted on the antenna, the pre-strain applied on the PDMS film is released and the liquid PDMS is then cured and (d) the labels are obtained by peeling off them from the substrate.

Due to the adhesion ability of the PDMS, the chip is directly connected to the Ag-NW circuit without any conductive adhesive. The equipment set-up for the RFID

reading experiment is shown in **Figure S3**. The chip used in the stretchable UHF RFID label is produced by Alien Technology LLC., USA, following the standard of EPC Class 1 Gen 2. The UHF reader is produced by East Core Smart Technology LLC, China, following the same standard. The characterization program is also produced by the East Core company, with the version of UHF V 1.8. During the test, the UHF reader was set to work at the reading speed of 20 times per second. The information in the chip was then shown on the computer screen.

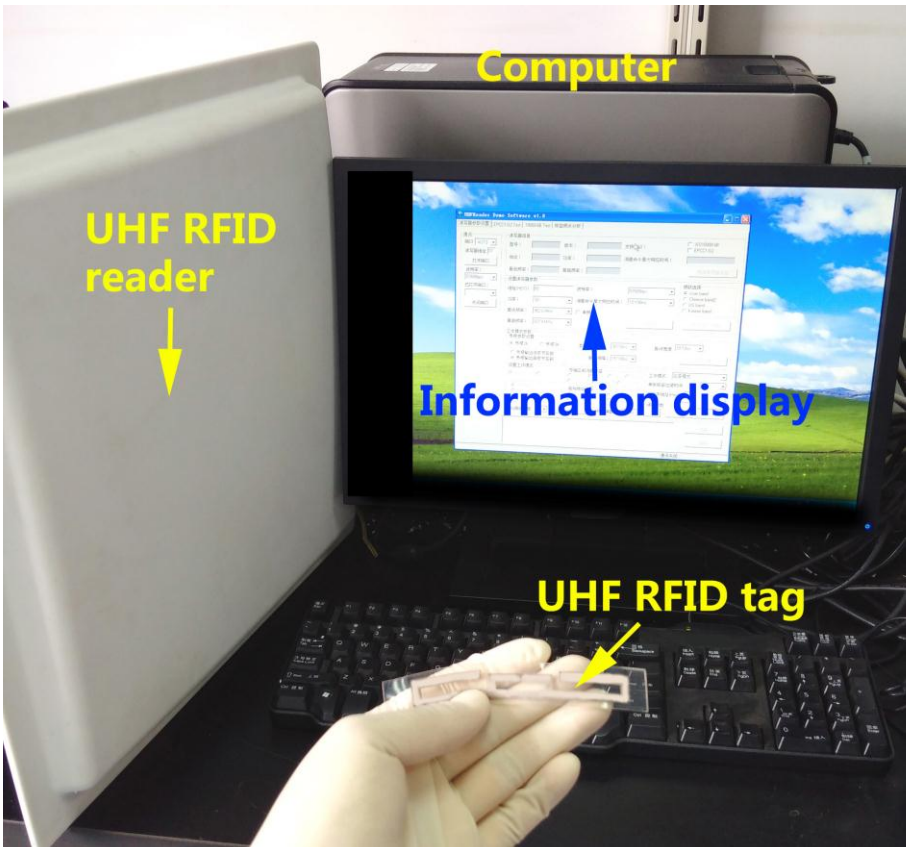

Figure S3. Digital photo of the equipment setup for the RFID reading experiment.

3. Laundry experiment

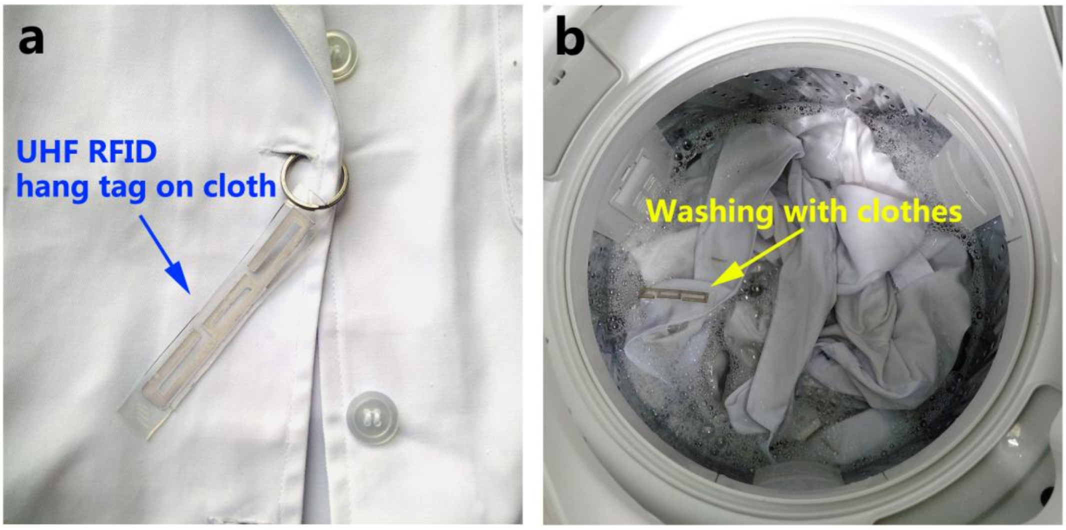

Figure S4. (a) Digital photograph for the stretchable UHF RFID label as a hang tag on a cloth and (b) digital photograph for washing of the RFID label with the clothes in a fully automatic washing machine.

Figure S4a and **S4b** show the digital photographs for the stretchable UHF RFID label working as a hang tag on a cloth and washing together with clothes in a Royalstar RB8018S impeller type fully automatic washing machine, respectively. The laundry experiment was conducted using the standard washing process. Each washing process includes one time of washing, two times of rinsing and three times of spin drying. Each process takes about 40 min. The RFID label was washed with other 5-7 clothes and the laundry water is set at room temperature. After each laundry, the label was dried naturally before taking conductivity and reading distance tests.
